# Xanthates As Useful Probes for Testing the Active Sites of Cytochromes P450 4A11 and 2E1

**DOI:** 10.3389/fphar.2017.00672

**Published:** 2017-09-22

**Authors:** Tsveta Stoyanova, Iglika Lessigiarska, Momir Mikov, Ilza Pajeva, Stanislav Yanev

**Affiliations:** ^1^Institute of Neurobiology, Bulgarian Academy of Sciences Sofia, Bulgaria; ^2^Institute of Biophysics and Biomedical Engineering, Bulgarian Academy of Sciences Sofia, Bulgaria; ^3^Department of Pharmacology, Toxicology and Clinical Pharmacology, Faculty of Medicine, University of Novi Sad Novi Sad, Serbia

**Keywords:** xanthates, cytochrome P450’s 4A11 and 2E1, fatty acids metabolism, molecular modeling, docking

## Abstract

Xanthates (alkyl or aryl derivatives of dithiocarbonic acid) have been shown to be selective mechanism-based inactivators of cytochromes P450 2B1/2B6 and 2E1 due to covalent binding of a reactive intermediate to apoprotein after double hydrogen abstraction at α-carbon atom, suggesting interaction of the xanthate dithiocarbonic head with the enzyme heme. The structures of xanthates with a long alkyl chain are similar to the fatty acids. Saturated fatty acids (FA) such as lauric acid (LA), are metabolized by different cytochrome P450 isoforms to ω- and (ω-1)-hydroxy products, in humans done by CYP4A11 and CYP2E1, respectively. In the present study we aimed at elucidating the possible interactions of xanthates with two cytochrome P450 isoforms CYP4A11 and CYP2E1 involved in the metabolism of the FA. Our experiments showed that LA-ω-hydroxylation by CYP4A11 is inhibited in a competitive manner by xanthates with long alkyl chain (C12-xanthate being the most potent inhibitor). On the other hand LA-(ω-1)-hydroxylation reaction by purified CYP2E1 is inactivated by a mechanism-based type. The suggested differences in the interactions of C12-xanthate with the two cytochrome P450 isoforms were investigated by molecular modeling using docking approach. The results suggested that in CYP2E1 active site C12-xanthate coordinates to the heme with its most vulnerable dithiocarbonic head leading to a mechanism-based inactivation. In CYP4A11 xanthate alkyl chain is exposed to the heme, thus, a potenial ω-hydroxylated xanthate product could be formed, which could inhibit in a competitive manner the hydroxylation of LA. The observed differences of xanthates interactions with the active sites of the two similar cytochrome P450 isoforms (CYP4A11 and CYP2E1) involved in the metabolism of FA, which lead to different changes in the enzyme activity, suggest that xanthates can be used as probing tools for analyzing enzyme active sites when exploring useful and selective compounds influencing FA homeostasis.

## Introduction

Fatty acids metabolism is related to many physiological and pathological processes like diabetes, gene expression (Jump et al., 1999), lipid peroxidation (Gueraud et al., 1999), regulation of vascular tone (Nguyen et al., 1999), immune system (Calder, 1999), hypertension (Engler et al., 1999), inflammation and cancer (Johnson et al., 2015).

Saturated FAs such as LA, are metabolized by CYP-dependent reactions to ω- and (ω**-**1)-hydroxy products. Enzyme activity tends to increase with fatty acid chain length [the activity is higher for myristic (C10) and lauric (C12) acids] and the ratio of ω-hydroxylase to (ω-1)-hydroxylase activity dependents on the particular substrate and P450 isozymes.

The CYP isozymes involved in the FAs metabolism are mainly from 4A to 2E families. They have different species and organ distribution: human 4A9, 4A11, 4A12, 4F2 (liver and kidney) and 2E1 forms, rat 4A1, 4A2, 4A3, and 4A8 forms (liver, kidney, and brain) (Roman, 2002). Human 4A11 metabolizes LA predominantly on ω-place (Powell et al., 1996), while 2E1 metabolizes LA on ω-1 place (Castle et al., 1995). The human liver 4F2 is mainly involved in arachidonic acid ω-hydroxylation (Powell et al., 1998).

Different potassium salts of alkyl or aryl derivatives of dithiocarbonic acid (xanthates) have been shown to be selective mechanism-based inactivators of cytochrome 2B6 and 2E1 by covalent binding of reactive metabolites to enzyme protein after splitting the chemical bond between the first carbon atom and oxygen. Other CYP isoforms tested were either inhibited in a non-competitive manner: strongly (CYP 1A1) or less effectively (CYP 2D6 and 2C9) or were without activity changes (CYP 3A2 and 3A4) (Yanev et al., 1999).

The structures of the xanthates with long alkyl chains are similar to the FA, which are hydroxylated with highest rate by CYP 4A and 2E1 isoforms (Powell et al., 1996). The aim of this study is to investigate the interactions of different xanthates with these CYP isoforms involved in FA hydroxylation. The experiments could provide additional information to explain better the mechanism of xanthates interactions with the CYP’s isoforms.

## Materials and Methods

### Chemicals

**FIGURE 1:**
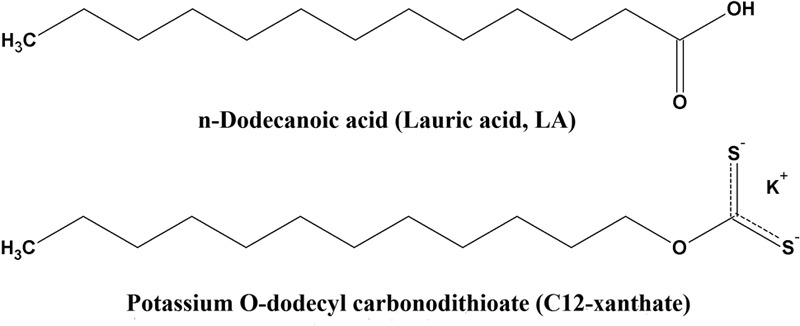
Chemical structures of lauric acid (LA) and potassium *O*-dodecyl carbonodithioate (C12-xanthate).

Various xanthates potassium salts (ROCS2K, where R is alkyl group with C3, C8, C10, C12, and C14 carbon atoms; for example, C3 = propylxanthate = CH_3_CH_2_CH_2_COS_2_^-^K^+^) were synthesized as previously described by Rao (1971). Compounds *O*-tricyclo[5.2.1.02,6]dec-9-yl-dithiocarbonate (D609), 7-Ethoxy-4-(trifluoromethyl)coumarin (7-EFC), 7-hydroxy-4-(trifluoromethyl)coumarin (HFC), LA, 12-HDA, HPA, PDAM and nicotinamide adenine dinucleotide phosphate, reduced (NADPH), were purchased from Sigma–Aldrich Chemical Co. (St. Louis, MO, United States). The structures of LA and C12-xanthate are shown in **Figure 1**.

### Enzyme Sources

Purified human CYP 4A11 (CYP content 1 nmole/ml) and bacteria expressed rabbit CYP 2E1 (cyrochrome P450 content 9 nmoles/ml) were obtained from Gentest Corp. (Woburn, MA, United States).

### Enzyme Activity

In order to study the possible mechanisms of enzyme–inhibitor interactions, we followed the classical experimental protocol for mechanism-based inhibitors: (i) pre-incubation at different concentrations of the xanthates with an enzyme source in a buffered medium fortified with NADPH (primary mixture); (ii) measurement of the remaining enzyme activity after different time intervals of incubation by starting the corresponding secondary reaction – LA-hydroxylation for CYP4A11 and 7-EFC-O- deethylation for CYP2E1.

#### Primary Mixture

The 200 μl reaction mixture consists of 0.1 M Tris–HCl buffer, pH 7.4; 5 mM MgCl_2_; aliquots of 4A11 supersomes containing 15 pmoles P450 or 320 pmoles 2E1 P450, as previously described (Yanev et al., 1999). The xanthates were tested at final concentrations of 100 μM (for 4A11) or C12-xanthate at final concentrations from 50 to 600 μM (for 2E1). The reaction was started with 10 μl NADPH (from 100 mM); after different time intervals of incubation at 37°C aliquots were taken for the secondary reaction.

#### Secondary Reaction

**Lauric acid**ω**-hydroxylation** by CYP 4A11 was tested following the procedure described by Mason et al. (1994) with some modifications.

The 200 μl reaction mixture consists of 0.1 M Tris–HCl buffer, pH 7.4, 5 mM MgCl_2_, 5 mM NADPH, 125 μM LA. The reaction was started with 40 μl aliquot taken from the primary mixture (3 pmol P450 4A11) at 0, 10, 20, and 30 min and incubated at 37°C for 20 min. The reaction was stopped with 1 ml cold ethyl acetate. After evaporation, the product of reaction 12-HDA in the dry extract was determined after coupling with PDAM by HPLC with fluorescent detection, using HPA as an internal standard.

**7-Ethoxytrifluoromethylcoumarin O-deethylation (7-EFC)** activity of CYP 2E1 was determined according to the method of Deluca et al. (1988).

Aliquots from the primary mixture (40 pmol P450 2E1 in 25 μl) were taken at different time intervals and were added into a final volume of 1 ml of a secondary reaction mixture containing 0.1 mM 7-EFC, 40 mg BSA/ml, and 0.2 mM NADPH in 50 mM potassium phosphate buffer (pH 7.4) (Buters et al., 1993). After incubation at 30°C for 15 min the reaction was stopped by adding 334 μl of ice-cold acetonitrile. The fluorescent product (HFC) was measured on a Perkin-Elmer MPF-44 spectrofluorometer with excitation at 410 nm and emission at 510 nm. The rate of HFC formation was calculated from a standard curve.

### Inhibitory Effect of C12-xanthate on LA-hydroxylation by CYP4A11

The inhibitory potency of C12-xanthate on the LA-ω-hydroxylation activity of CYP 4A11 was tested by incubating the enzyme along with various concentrations of C12-xanthate (10, 25, and 50 μM) and different concentrations of LA (from 10 to 100 μM).

## Molecular Modeling (Docking)

The structure of C12-xanthate was built and optimized with MMF94 force field using MOE software v. 2014.09 (Chemical Computing Group^1^).

The structure of CYP 2E1 was taken from the Protein Data Bank (PDB ID 3KOH, co-crystallized with omega-imidazolyl octanoid acid). The enzyme structure was prepared with *Structure Preparation Tool* in MOE including *3D Protonate* to assign the correct ionization states and to position hydrogen atoms in the X-ray protein structure. Subunit A of 2E1 PDB 3KOH structure was used, the water molecules were deleted (five molecules), because they were outside the enzyme active site.

The structure of CYP 4A11 was taken from Swiss Model repository (ID Q02928, downloaded on 8 December 2013). The structure did not contain heme. According to the homology models of 4A11 published by Chang and Loew (1999) and Lewis and Lake (1999) the Fe atom of the heme is attached to Cys457 [corresponding to Cys400 in CYP102 (P450BM3) used as a template in the models]. According to Hoch and Ortiz de Montellano (2001) the heme is connected to 4A11 via an ester link to Glu321, which is predicted to be within the active site (Chang and Loew, 1999). In order to place the heme in the structure of 4A11, 4A11 was aligned with *Superpose tool* in MOE to the structure of 2E11 according to the cysteine residues connected to the heme (Cys437 in 2E1 and Cys457 in 4A11), and the heme was placed in the 4A11 structure with the same orientation as in 2E1 structure. The protein structure was optimized with Amber12:EHT force field.

Docking into the active sites was done with *Docking tool* in MOE. The default settings were used in the docking protocol: London dG scoring function for Rescoring (1), GBVI/WSA dG scoring for Rescoring (2), Triangle Matcher placement method, and Forcefield refinement method (MMF94 forcefield). The docking poses were ranked by the scores from the GBVI/WSA binding free energy calculation. The best 10 docking poses (poses with the lowest scores) of C12-xanthate were kept and analyzed. For docking in 2E1 the active site was defined as all amino acid residues located within 4.5 Å (a default MOE setting) from the heme and the ligand (omega-imidazolyl octanoid acid) in the crystal structure of the enzyme, and including the heme. For 4A11 the active site was chosen by all amino acid residues located within 4.5 Å from the heme, adding the amino acid residues listed as a part of the active site according to Chang and Loew (1999), and including the heme. In order to avoid bias due to the way the heme was placed in the structure of 4A11, docking without heme was also done. In this case the active site was defined by the amino acid residues listed as a part of the active site (Chang and Loew, 1999).

## Statistics

Non-linear and linear regression analysis to calculate enzyme kinetics parameters was done using GraphPad Prism 6.0 software. Paired *t*-test was applied for the statistical comparison of the slope values of residual enzyme activity data. The values are mean of three replicated measurements.

## Results

### Effect of Different Xanthates on LA Metabolism by 4A11

**FIGURE 2:**
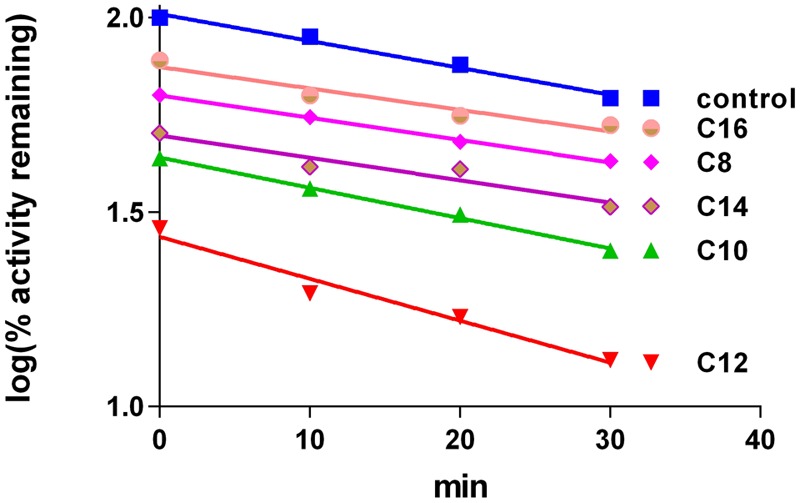
Lauric acid-hydroxylation by purified human CYP4A11. Time-dependent changes induced by different xanthates (100 μM in primary medium, 20 μM in secondery medium). The values are mean of three replicated measurements.

Incubation of different xanthates in concentration of 100 μM with 4A11 supersomes and NADPH for 30 min did not show any time-dependent inactivation pattern of LA hydroxylation in the secondary incubation medium. The slopes of the line describing the decay of CYP 4A11 enzyme activity in xanthates’ samples (-0.006928 ± 0.0005986 for C12 samples) after different time intervals of pre-incubation were not significantly different from that of the control samples (-0.01078 ± 0.001414) (*p* = 0.066, by paired *t*-test). The samples for testing LA-hydroxylation could not be diluted to a greater extent because the amount of the enzyme in the aliquot sample (3 pmoles CYP4A11) should be sufficient to perform an optimal enzymatic reaction in the secondary mixture. Thus, the final xanthate concentration of 20 μM in the secondary reaction medium is high enough to lead to different degrees of inhibition by xanthates with different alkyl chain lengths as C8, C10, C12, C14, and C16 at zero time-point (**Figure 2**). The most potent inhibitor was found to be C12, xanthate with the most similar chemical structure to the natural substrate LA (**Figure 1**). No inhibitory effect was observed by C3-xanthate having short alkyl chain length, and D609, a xanthate with cyclic aryl substitution (data not shown).

### Inhibition of CYP 4A11 LA-ω-hydroxylation by C12-xanthate

**FIGURE 3:**
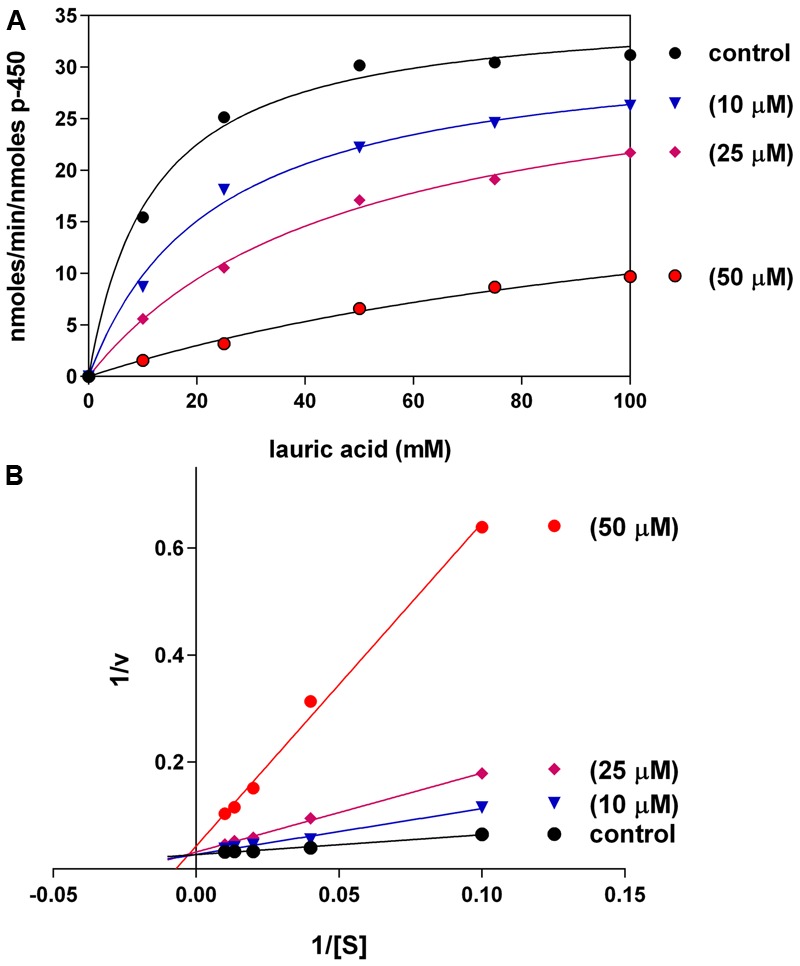
Effects of different concentrations of C12-xanthate on CYP4A11 LA-hydroxylation **(A)** Michaelis–Menten kinetics (non-linear regression analysis); **(B)** Lineweaver–Burk plot (linear regression analyis). The values are mean of three replicated measurements.

C12-xanthate inhibited LA-ω-hydroxylation in 4A11 supersomes when incubated in different concentrations (10, 25, and 50 μM) (**Figure 3A**). The hydroxylation of LA by supersomes followed Michaelis–Menten kinetics with *K*m (=11.8 μM) and *V*max (35.8 nmoles/min/nmoles P450). The type of C12 inhibition was found to be competitive (**Figure 3B**). The calculated *K*i was 9.0 ± 1.0 μM. At the higher concentrations used (50 and 100 μM) the mechanism switched to mixed type inhibition.

### Effect of C12-xanthate on 2E1 Supported 7-EFC Metabolism

**FIGURE 4:**
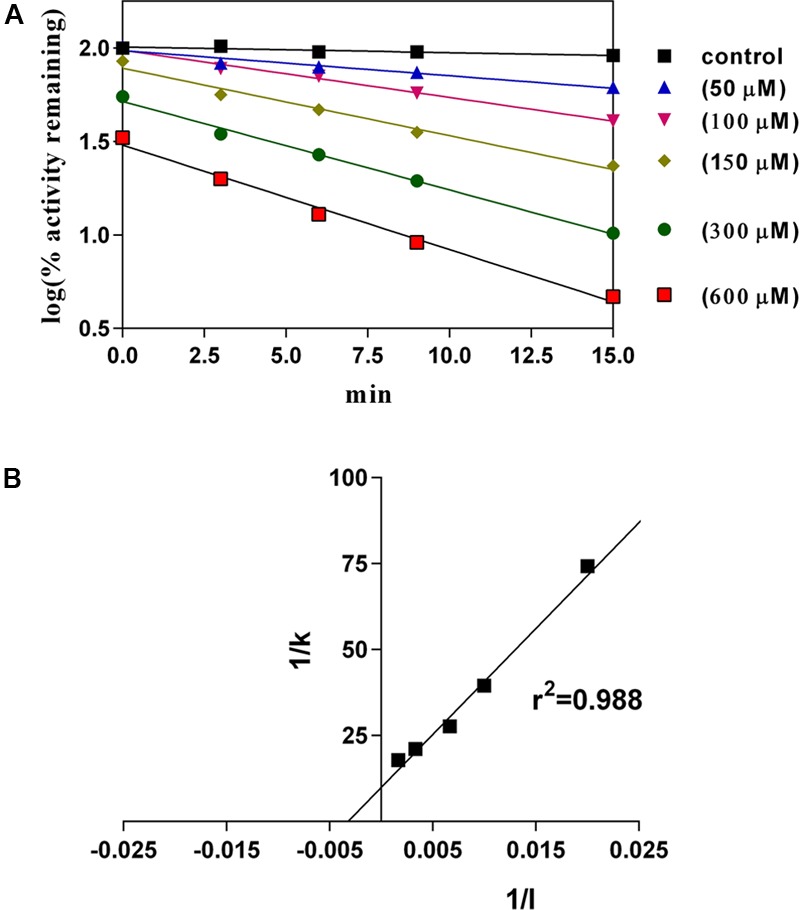
Time- and concentration-dependent changes induced by C12-xanthate (50 to 600 μM in primary medium) on EFC-deethylaion by purified rabbit CYP2E1 (the values are mean of three replicated measurements). **(A)** log plot of enzyme activity remaining; **(B)** Calculation of inactivation constants by ploting 1/inhibitor concentration (1/I) to 1/slope (1/k) (linear regression analysis).

C12-xanthate in a concentration range from 50 to 600 μM affected the 2E1 supported 7-EFC reaction as a typical mechanism-based inactivator (**Figure 4A**). The calculated rate constant (*k*_inact_), half-live of inactivation (*t*_1/2_) and inactivation constant (*K*_I_) were 0.1 min^-1^, 7 min and 312 μM, respectively (**Figure 4B**).

### Molecular Modeling

The score results from the docking of C12 to 2E1 and 4A11 are presented in **Tables 1, 2**, respectively. E_conf is the conformation energy of C12. E_place is the score from the placement stage in docking; it reflects the energy of placement of the ligand in the active site. The interaction of the ligand with the enzyme is evaluated by the score *S*-value; lower S corresponds to a stronger interaction (MOE v. 2014.09).

**Table 1:** Docking of C12 to CYP **2E1.**

	*S*	E_conf	E_place
min	-13.57	-59.81	-96.88
Pose on **Figure 5**	-13.57	-49.94	-56.10
max	-10.28	-48.92	-47.51

*S, score of ligand–enzyme interactions; E_conf, conformation energy of C12; E_place, score from the docking placement stage.*

**Table 2:** Docking of C12 to CYP **4A11**.

	*S*	E_conf	E_place
min	-8.59	-65.88	-67.48
Pose on **Figure 6**	-8.59	-62.73	-62.86
max	-7.80	-55.64	-47.61

*S, score of ligand–enzyme interactions; E_conf, conformation energy of C12; E_place, score from the docking placement stage.*

**FIGURE 5:**
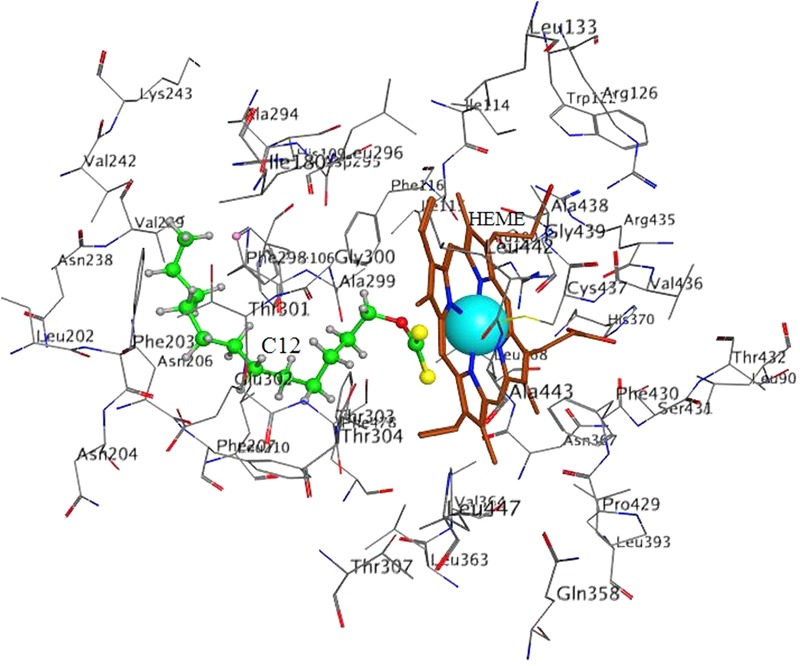
Docking of C12 to CYP 2E1. C12 oriented with its dithiocarbonic group toward the heme. Atom colors denote: C in C12 – **green**, C in heme – **brown**, C in protein – **gray**, O – **red**, S – **yellow**, N- **dark blue**, Fe – **light blue**.

**FIGURE 6:**
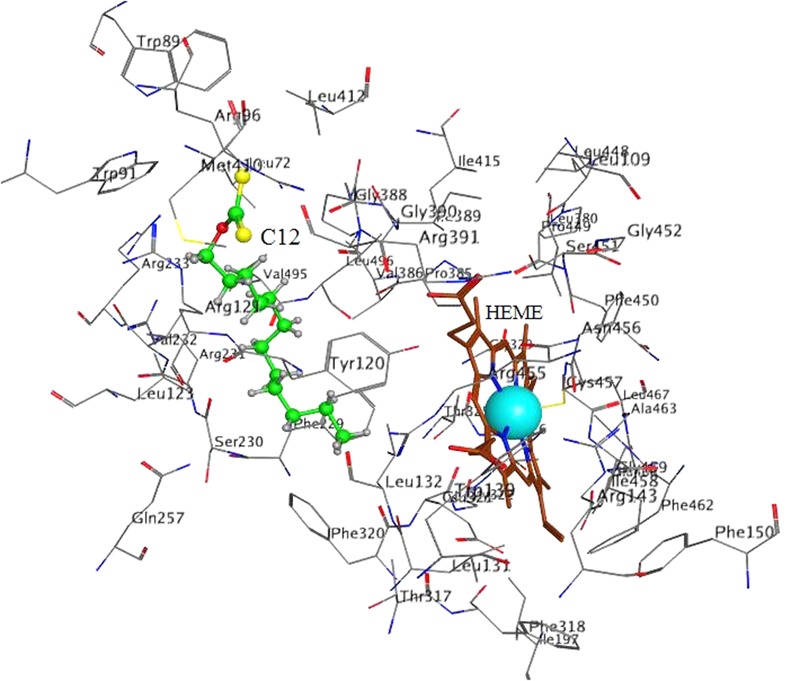
Docking of C12 to CYP 4A11. C12 oriented with its alkyl chain toward the heme. Atom colors as on **Figure 5**.

The best poses (with the lowest *S*-values) in 2E1 and 4A11 are presented in **Figures 5, 6**, respectively.

In **Tables 1, 2** the minimum and maximum values for the best 10 docking poses of C12, and the values for the best poses (**Figures 5, 6**), are presented. The best 10 poses had similar *S*-values with difference less than 3.5 kcal/mol.

In 2E1 in all 10 poses C12 was oriented with the dithiocarbonic group toward the heme (**Figure 5**). Thus, the 10 poses with the strongest interactions with the enzyme were oriented with the dithiocarbonic group toward the heme, suggesting that this orientation of C12 in 2E1 is more preferable.

In 4A11 C12 was oriented with the alkyl chain toward the heme (**Figure 6**) in 9 poses, and with the dithiocarbonic group toward the heme in one pose. Because in 9 out of 10 poses with the strongest interaction C12 was oriented with the alkyl chain toward the heme, this orientation in 4A11 could be considered as more preferable than the orientation with the dithiocarbonic group toward the heme, in contrast to the C12 orientation in 2E1.

**FIGURE 7:**
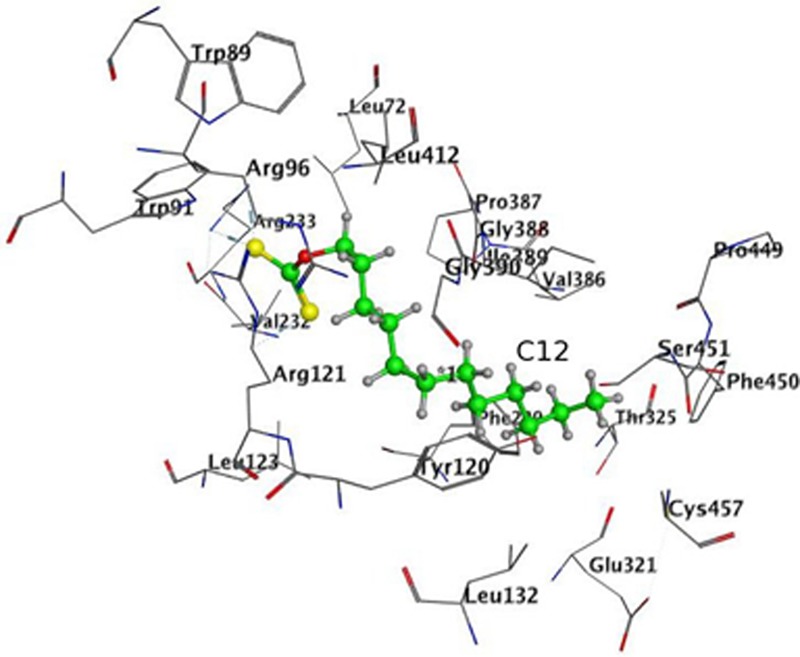
Docking of C12 to CYP 4A11 without heme. C12 oriented with its alkyl chain toward Cys457. Atom colors as on **Figure 5**.

In order to avoid bias due to the procedure of placement of heme in the structure of 4A11, docking in 4A11 without heme in the enzyme was done. The results are shown in **Figure 7** and **Table 3**. The docking results with a heme in the active site were reproduced. Again, minimum and maximum values for the best 10 docking poses of C12, and the values for the best pose, are presented in **Table 3**. C12 was oriented with the alkyl chain toward Cys457 (**Figure 7**) in all 10 poses, suggesting again that this orientation could be more preferable. The repeated docking runs reproduced these results.

**Table 3:** Docking of C12 to CYP **4A11** without heme.

	*S*	E_conf	E_place
min	-9.16	-62.77	-66.05
Pose on **Figure 7**	-9.16	-62.77	-66.05
max	-8.25	-55.55	-48.70

*S, score of ligand–enzyme interactions; E_conf, conformation energy of C12; E_place, score from the docking placement stage.*

## Discussion

The hydroxylation of LA was affected by the xanthates in a different manner depending on the CYP isoform involved in the reaction.

LA-ω-hydroxylation by CYP4A11 was inhibited in a competitive manner by the xanthates with a long alkyl chain (C12 being the most potent inhibitor).

Xanthates are relatively good substrates for CYP2E1 (shown in these experiments for C12-xanthate as well as in already published data for other xanthates (Yanev et al., 1999) leading to the quick inactivation of the enzyme activity when tested with 7-EFC. The predominantly LA-(ω-1)-hydroxylation reaction by purified CYP2E1 is inactivated by a mechanism-based type. The inactivation constant for C12 measured in these experiments is lower than that for C8 found in our previous study, 312 and 60 μM, respectively (Yanev et al., 1999).

It is unlikely the observed difference in the xanthates interaction with the two CYP isoforms to be due to the use of P450s derived from different species (human CYP4A11 and rabbit CYP2E1). The enzyme kinetic parameters suggest very small differences in the chlorzoxazone 6-hydroxylase activity of CYP2E1 among different species (human, rat, rabbit, mouse, dog, and monkey) (Bogaards et al., 2000). Additionally, similar activity of human and rabbit 2E1 related to the metabolism of different FA and alcohols has been observed (Bell-Parikh and Guengerich, 1999). In this respect the CYP2E1 subfamily was declared as a “subfamily in which species extrapolation appears to hold quite well” (Guengerich, 1997). Thus, large differences in the way of interaction of xanthates with the same enzyme isoform derived from different sources could not be expected.

There are some similarities and also some differences between the active sites of 4A11 and 2E1 forms.

The compounds, which have been found to inhibit the 4A11-dependent LA-hydroxylation, possessed not only structural similarity with the natural substrate (long alkyl chain) but also some vulnerable chemical groups. Thus, the 12-carbon terminal acetylenic fatty acid, 11-dodecynoic acid, is a potent mechanism-based inactivator (Muerhoff et al., 1989). Some ω-substituted FA (with imidazole or amino groups) have been shown to be powerful competitive heme-coordinating inhibitors (Lu et al., 1997). The QSAR analysis of interactions of different substrates and inhibitors of 4A11 with the enzyme active site suggested not only a high dependence of the activity on the length of the molecule (around 14Å distance between the protein polar recognition binding site and the heme ferryl group) but also a high dependence on steric factors [only compounds with “straight” alkyl chain have a chance to coordinate the heme (Bambal and Hanzlik, 1996)]. This steric dependence could explain the fact that 4A11-dependent LA-hydroxylation takes place on the terminal carbon atom (ω-), the thermodynamically more unfavorable place.

The active site of 2E1 is similar to 4A11 but possesses a small “pocket” near the heme which hydrophobicity allows the LA to be hydroxylated preferably to (ω-1) place, the most energetically favorable place (Wang et al., 1995; Adas et al., 1999). That is why 2E1 could metabolize also large number of alcohols and carboxylic acids.

Our results demonstrate that xanthates with long alkyl chain (like C12-xanthate) are relatively good substrates for both CYP forms involved in the LA-hydroxylation. The xanthate metabolism by the two CYP isoforms leads to different changes in the xanthate molecule, which results in different changes in the enzyme molecule. Our molecular modeling results with C12-xanthate support the hypothesis that the differences may be due to the different ways of interaction with the P450 enzymes. According to the results the 2E1 active site allows C12-xanthate to coordinate to the heme with its most vulnerable dithiocarbonic head leading to the mechanism-based inactivation. In 4A11 the xanthate alkyl chain is exposed to the heme, thus, a potential ω-hydroxylated xanthate product could be formed, which could inhibits in a competitive manner the hydroxylation of LA. It is interesting to note that C12 inhibitory constant for 4A11 (9 ± 1.0 μM) is of the same order of magnitude with LA-ω-hydroxylation Michaelis–Menten constant found in human liver microsomes: 13.6 μM, 22 μM (Clarke et al., 1994) or 48.9 μM (Powell et al., 1996) and in purified 4A11: 11 μM (Gainer et al., 2005; Kim et al., 2014) and 12.5 μM (Songhee et al., 2009).

Can similar modes of interactions be suggested between the xanthates and other CYP isozymes from 4A family involved in FAs metabolism? Having in mind the unique covalently linked heme in this particular CYP family (Hoch and Ortiz de Montellano, 2001) it is important to consider to what extent the changes in enzyme activity by xanthates could be due to the changes in the ratio of covalent to free heme. In our preliminary experiments with purified rat isoforms 4A1 activity was not affected after incubation with xanthates with longer alkyl chain. On the other hand, the activities of 4A3 and 4A8 were slightly decreased after 30 min of incubation with C12 in a non-concentration dependent manner. This could be due to an observed increase in the amount of the free compared to the covalent heme attached to these particular isoforms after the xanthate interaction but not to the binding to the enzyme active side (Yanev and Ortiz de Montellano, 2002, Unpublished). Another possible explanation could be that there are some differences in the binding and substrate accessibility to the active site between human 4A11 and rat 4A forms as shown by Dierks et al. (1998).

Our results suggest an enzyme dependant manner of interaction of the investigated xanthates with CYP’s, and point out that these compounds could be useful probes for distinguishing between 4A11 and 2E1-dependent reactions when exploring useful and selective compounds influencing FA homeostasis.

## Author Contributions

SY and IP planned the works, analyzed results, and proposed explanations and wrote the paper. TS and IL carried out experiments and reported data. MM assisted in data review and proofing the manuscript.

## Conflict of Interest Statement

The authors declare that the research was conducted in the absence of any commercial or financial relationships that could be construed as a potential conflict of interest.

## Footnotes

1 https://www.chemcomp.com/

## Abbreviations

12-HDA: 12-hydroxydodecanoic acid
7-EFC: 7-ethoxytrifluoromethylcoumrin
7-HFC: 7-hydroxytrifluoromethylcoumrin
C12-xanthate: potassium *O*-dodecyl carbodithioate
CYP: Cytochrome P450
FA: fatty acids
HPA: 16-hydroxypalmitic acid
LA: lauric acid
PDAM: 1-Pyrenyldiazomethane
